# Measuring Central and Eastern Europe’s Socio-Economic Development Using Time Lags

**DOI:** 10.1007/s11205-015-0991-9

**Published:** 2015-05-26

**Authors:** Dominik Paprotny

**Affiliations:** Faculty of Civil Engineering and Geosciences, Delft University of Technology, Stevinweg 1, 2628 CN Delft, The Netherlands

**Keywords:** Time lags, Communist states, Social development, Health indicators, Economic indicators

## Abstract

This paper applies the ‘time lag’ method to a set of social and economic indicators, examining the development of Central and Eastern Europe since the first world war. Originally used to assess technology diffusion, this method allows comparison of levels of development between states and through a long period of time. It presents how many years have elapsed between achieving a certain level of development between countries. The results show that the countries of Central and Eastern Europe have only narrowly converged with a set of 23 highly-developed ‘benchmark’ states. Development in monetary terms (gross domestic product per capita) is the indicator where this region lags most. Employment structure, life expectancy or infant mortality show much smaller lags. Communist states were closest to the West in the 1960s–early 1970s and struggled thereafter. They are still mostly lagging more today than at their peak before transformation despite the progress achieved in absolute terms after the fall of centrally-planned economy.

**Electronic supplementary material:**

The online version of this article (doi:10.1007/s11205-015-0991-9) contains supplementary material, which is available to authorized users.

## Introduction

It is problematic to present indicators of development, well-being or quality of life in a comparative perspective over a long period of time. Whereas it is easy to show whether a country is progressing or not in absolute terms (for example, how life expectancy of its population is changing), it’s far more difficult to analyse whether it is progressing relative to another country. Is it converging or the opposite, diverging in relative backwardness? Suitable econometric methods have been developed, but they are used to assess convergence in whole groups of countries or regions, not individual states (Barro and Sala-i-Martin 1992; Monfort 2008). Recently, Jordá and Sarabia (2014) used such methods to analyse ‘beta’ and ‘sigma’ convergence of the United Nation’s Human Development Index. They found that, in general, countries converged in development since 1980, though they did it to a greater extent in education and health than in income. Those findings are also in contrast with several other studies, with indicated divergence in development (Mazumdar 2003; McGillivray and Pillarisetti 2004).

Proper measures of cross-country comparisons are scarcer. Eurostat (2014) uses a relative indicator of gross domestic product (GDP) per capita derived by dividing the value for a European Union (EU) member state by the EU average. By this measure, for example, Poland made significant progress between 2002 and 2012: its GDP per capita increased from 47 to 66 % of the EU average, similarly to other Central European member states. However, an application of such a method to other socio-economic indicators like life expectancy, infant mortality or employment structure, would be dubious at best.

A possible alternative is the ‘time lag’ concept. It was first properly described by Comin et al. (2008), who utilized it to analyse technology diffusion. For indicators that increase or decrease with progress over a long period of time, it is possible to calculate the time difference between achieving a certain value of an indicator. In other words, how many years a less-developed country is behind relative to a well-developed one. If life expectancy of a woman born in Romania in 2012 was 78.1 years, it is a level of development which was already sustained by Norway since 1976. Therefore, Romania lags Norway in female life expectancy by 36 years. Comin et al. (2008) limited their study to analysing technological indicators and GDP per capita, a few time points and a single country of comparison (the United States). Some other small-scale studies have also been carried out. Jovanovic (2009) incorporated calculations of time lags of electricity production in a study on life-cycles of technologies. French (2014) analysed lags in the adoption of medical technologies as an argument in a study on the relation between mortality and technology diffusion. Paprotny (2014) adopted the method in order to compare the relative progress of Poland with rich Western countries.

Here, I present the usefulness of the ‘time lags’ method for analysing social indicators and their change over time. The study was carried out on a particularly interesting example, i.e. Central and Eastern Europe (CEE) states. The whole region was tormented by the turbulent history of the twentieth century. Its economy was battered by two world wars, the Great Depression, the rise and fall of communist rule, civil conflicts and coup d’états (Aldcroft 1993). Most of CEE countries only gained (or regained) independence in the 1990s, a decade also marked by a painful transition from a centrally-planned to a free-market economy (Good and Ma 1999; Maddison 2001). At first, this shift impoverished the already poor CEE countries even further, but subsequently led to a remarkable economic expansion. Nevertheless, there is still a significant development gap between rich Western Europe states and the majority of CEE countries. The latter are generally considered as in ‘middle development’, with some aspiring to be regarded as highly developed, especially the Czech Republic and Slovenia, while others are struggling. Some could even be downgraded to the ‘low development’ category, like Ukraine and Moldova (Nielsen 2013). Inside the European Union it is a serious concern, since it creates tensions between member states about the financial support aimed at narrowing this gap. A low level of development of candidate states is seen as the most serious barrier for their accession to the EU.

The analysis of the region’s performance covers a time period between 1920 (shortly after the end of the Great War) and 2012. Before the war only a few CEE states were independent; moreover, pre-1914 statistical evidence is scarce. All countries of the region during that period are compared with a set of highly developed OECD members, dubbed henceforth as the ‘benchmark group’. Seven indicators representative for the whole time period were selected, dealing with different aspects of socio-economic development.

## Materials and Methods

In this section, the ‘time lag’ method will be presented in detail, as well as the selection of countries and indicators will be discussed, as these aspects are important for the analysis.

### The ‘Time Lag’ Method

The ‘time lag’ method is best illustrated by an example, as already noted in the introduction. Consider Poland, where male life expectancy at birth was 72.7 years in 2012. Norway surpassed that level in 1983 and it hasn’t fallen below that value as of 2012. Therefore, Poland lags Norway in male life expectancy by 29 years. Mathematically, let *X*_*j,t*_ be the value of an indicator in CEE country *j* in year *t* and {*X*_*k,s*_} the set of observations (indexed by *s*) for benchmark country *k*. The year 1983 from our example will be $\bar{s}_{k}$, which is the year which country *k* last recorded a level equal to or lower to *X*_*j,t*_:

1 $$\bar{s}_{k} = \mathop {\arg \hbox{max} }\limits_{s \in S} \left\{ {\left. s \right|X_{k,s} \le X_{j,t} } \right\} + 1$$

where *S* is the entire set of observations for country *k.*^1^ The time lag (say *ω*) for country *j* will, therefore, be:

2 $$\omega_{j} = t - \left( {\frac{1}{K}\sum\limits_{k = 1}^{K} {\bar{s}_{k} } } \right)$$

where *K* is the number of benchmark countries. In the aforementioned example, the time lag of Poland in 2012 derived from Eq. (2) will be 23.1 years. Time lags *ω* have positive values, if a CEE country is less developed than the benchmark and negative if it outperforms them on average. However, the presented example and its formalization are true for indicators with values increasing over time. For variables decreasing with progress—like infant mortality—the time lag is calculated the other way round. Thus, if infant mortality in Bulgaria in 1990 was 14.8 deaths per 1000 live births, this parameter was below that level during 1966–1990 in Iceland (time lag of 24 years). Consequently, Eq. (1) becomes:

3 $$\bar{s}_{k} = \mathop {\arg \hbox{max} }\limits_{s \in S} \left\{ {\left. s \right|X_{k,s} \ge X_{j,t} } \right\} + 1$$

while Eq. (2) remains unaltered.

As can be seen in Eq. (2), an arithmetic average of time lags for individual benchmark countries is used. An alternative would be to calculate a weighted average, but then a few countries would dominate the comparison: the United States and Japan constitute half of the benchmark group’s population between themselves. Each country had its individual path to prosperity, therefore is using an arithmetic average which preserves the importance of smaller countries in the comparison.

### Countries

There are a total of 23 entities in the CEE countries group: Albania, Belarus, Bosnia and Herzegovina, Bulgaria, Croatia, the Czech Republic, Estonia, Hungary, Latvia, Lithuania, Macedonia, Moldova, Poland, Romania, Russia, Serbia with Montenegro,^2^ Slovakia, Slovenia, Turkey and Ukraine. The former states of Czechoslovakia (defunct since the end of 1992), the Soviet Union (USSR, dissolved in 1991) and Yugoslavia (broken up during 1991–1992) are also included. All available statistical data from 1920–2012 were collected, even if a state was not independent at a given moment of time. For now-defunct countries, aggregates were created using data for their successor states.^3^

The benchmark group is also comprised of 23 countries: Australia, Austria, Belgium, Canada, Denmark, Finland, France, Germany, Greece, Iceland, Ireland, Italy, Japan, Luxembourg,^4^ the Netherlands, New Zealand, Norway, Portugal, Spain, Sweden, Switzerland, the United Kingdom and the United States. All of them are OECD members and are among the most developed countries in the world (Nielsen 2013). As can be seen Table 1, all countries (except Portugal) constitute 22 of the 30 most developed states in 2012, using one of the popular indicators, the Human Development Index (HDI). Several other states were considered, but omitted. South Korea (ranked 12 by HDI) and Taiwan (not ranked) have adequate statistical data, but they were considerably poorer than the CEE states up until the 1970s. Their inclusion would therefore impact the results. Some other countries from the top of the HDI list lacked sufficient amount of data suitable for this study, like Israel (ranked 16), Singapore (18), Cyprus (31) and Malta (32).

**Table 1:** Benchmark and CEE countries ranked by the 2012 Human Development Index

Benchmark countries			CEE countries		
HDI ranking	Country	HDI value	HDI ranking	Country	HDI value
1	Norway	0.955	21	Slovenia	0.892
2	Australia	0.938	28	Czech Republic	0.873
3	United States	0.937	–	*Czechoslovakia*	0.862^a^
4	Netherlands	0.921	33	Estonia	0.846
5	Germany	0.920	35	Slovakia	0.840
6	New Zealand	0.919	37	Hungary	0.831
7	Ireland	0.916	39	Poland	0.821
8	Sweden	0.916	41	Lithuania	0.818
9	Switzerland	0.913	44	Latvia	0.814
10	Japan	0.912	47	Croatia	0.805
11	Canada	0.911	50	Belarus	0.793
13	Iceland	0.906	55	Russia	0.788
15	Denmark	0.901	56	Romania	0.786
17	Belgium	0.897	57	Bulgaria	0.782
18	Austria	0.895	–	*Yugoslavia*	0.779^a^
20	France	0.893	–	*Serbia with Montenegro*	0.770^a^
21	Finland	0.892	–	*USSR*	0.753^a^
23	Spain	0.885	70	Albania	0.749
25	Italy	0.881	78	Ukraine	0.740
26	Luxembourg	0.875	78	Macedonia	0.740
26	United Kingdom	0.875	81	Bosnia and Herzegovina	0.735
29	Greece	0.860	90	Turkey	0.722
43	Portugal	0.816	113	Moldova	0.660

^a^Aggregate calculated from population-weighted successor states’ HDI

### Indicators

Choosing proper indicators for the study was difficult, as they had to fulfil several conditions. In general, indicators had to be comparable between states, i.e. their definitions, completeness and reliability of reporting had to be relatively consistent between states and throughout the study’s timeframe. At the same time, they should represent genuine progress instead of a local geographical, cultural and administrative reality. Furthermore, they should be representative for the whole timespan of the study.

Examples of popular indicators not fulfilling these criteria are easy to point out. Percentage of the workforce employed in industry, as well as production statistics of selected goods, like iron and steel would have been useful when analysing the nineteenth century, but are no longer so, as this branch of economy is has been declining during the past century in advanced countries (Maddison 2001). Notwithstanding the size of the industrial and services sectors, which vary immensely between countries. Urbanization (percentage of people living in urban localities) has very much limited comparability between states because of different definitions (United Nations 2013). Efficiency of agriculture is dependent on climate and cultivation methods as much as on financial resources. Electricity usage varies according to climate and economic structure; in CEE countries, it has actually declined since the 1980s, despite large economic expansion (Eurostat 2014). Educational indicators such as literacy and primary/secondary school enrolment have already reached near 100 % several decades ago in all countries used in this study, though the indicator could be useful when analysing the least developed states. Meanwhile, tertiary education enrolment or attainment is very diversified between advanced countries (Barro and Lee 2013, UNESCO 2014). Usage levels of several technologies, both modern (the internet) and obsolete (telegraph) are not representative for the whole 1920–2012 timeframe. Fertility rates, declining throughout the world due to “demographic transition” (Reher 2004), are very diversified between advanced states—and usually higher than in CEE states. Finally, more modern measures of quality of life and well-being, often survey-based (de Smedt 2013), are unavailable for longer time periods.

With so many constrains in mind, only seven indicators were chosen: GDP per capita, employment in agriculture relative to total employment, male and female life expectancy, infant mortality rate, telephone and passenger car usage. Each measure has its specifics and rationale behind it, further explained in the text below. However, the biggest issue was to complete two databases for CEE countries and the benchmark countries. The former included all available data from 1920–2012, while the latter had a much broader timespan in order to provide proper comparison, as the CEE data had to be often juxtaposed with nineteenth century data for advanced economies. The primary sources of data were the websites and publications of the national statistical institutes (NSEs) of each country analysed.^5^ Other sources, which include databases of international institutions and research papers, are listed in “Appendix”.

*GDP per capita* is by far the most popular measure of development and well-being, even though highly criticized (Stiglitz et al. 2009). GDP, the monetary representation of all goods and services produced during a year in a country is in use since the 1930s, though estimates go as far back as the antiquity (Maddison 2003). Gross domestic product is measured here in 2005 purchasing power parities, which means that the value of GDP for all countries was adjusted to a single price level (namely, that of the United States in 2005) for a better comparability of real output. All GDP values have additionally been adjusted to the exchange rates and value of the US dollar in the year 2013. Using different time moments for calculating the parities and constant prices would change the exact values of lags, though would have no impact on the underlying trend.

*Employment in agriculture* is a measure of modernity of the economy. In contrast to employment in industry and services it is less dependent on country specifics and consistently decreases with progress. In general, this indicator covers persons employed in agriculture (including fishery and forestry) as a percentage of total employment, though for some countries and time periods the definition is broader, covering the total economically active population.

*Infant mortality rate* is by far the most reliable indicator of quality and accessibility of health care. It is defined as the number of deaths of infants during the first year of life (stillbirths excluded) per 1000 live births during the reference year. This definition is generally consistent between states, though historical data for some states have lower reliability due to under-reporting (if an infant died before its birth was reported to the authorities, it was classified as a stillbirth).

*Life expectancy* is one of the most important measures of quality of life and development. Here, life expectancy at birth is analysed separately for men and women. It is calculated in fairly similar manner in all countries, though its reliability varies depending on the quality of mortality and population structure data. In this indicator, mortality rates by age are used to calculate the average number of years individuals from a cohort are expected to live, assuming no changes in mortality over time.

*Telephone usage* is defined as the number of fixed telephone lines and mobile phone subscriptions per 100 persons. Access to this technology is a good indicator of socio-economic progress in a country, though in recent years the rapid development of mobile telephony made it ubiquitous around the world. Also, historical data for some countries refer to number of devices rather than the number of mainlines, exaggerating the figures somewhat.

*Passenger car usage* is a decent descriptor of development level, as cars are expensive goods and a hallmark of wealth. The indicator is defined as the number of registered passenger cars per 100 persons. The exact definition of a passenger car varies by country, but generally refers to vehicles capable of carrying no more than nine passengers and excludes tractors, motorbikes, buses, trucks or special-purpose vehicles.

**Fig. 1:**
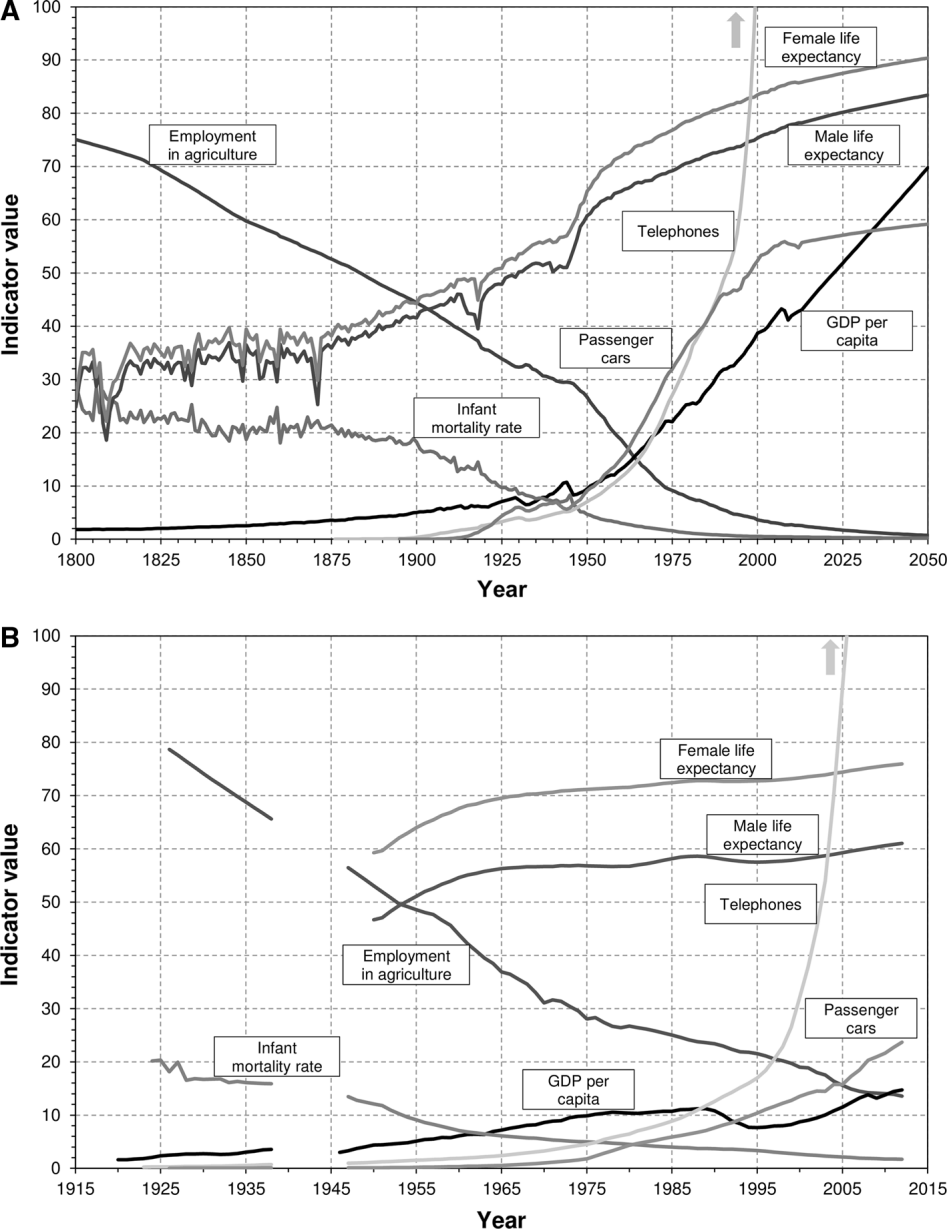
Development indicators for the benchmark group (1800–2050) and CEE countries, (1920–2012), all population-averaged. Employment in agriculture as % of total employment; GDP per capita in US$, 2005 PPPs and 2013 prices; life expectancy at birth in years; infant mortality rate per 100 live births; passenger cars and telephones per 100 persons

For all benchmark countries and the indicators a dataset covering the years 1800–2050 was compiled, except data for telephones (1876–2050) and cars (1895–2050), as they were only invented in the end of nineteenth century. Such a large timeframe was needed to provide adequate comparison to CEE states. As mentioned above, the dataset have a complete annual coverage, though it required several interpolations and an extension of timespan of the original data. Extrapolations to the past were made using the assumption that the values for a country with missing data run parallel to a geographically proximate country, for which data are available (e.g. change in infant mortality for Belgium before 1841 corresponded to the indicator’s variation in France). Data for infant mortality and life expecÀcy for 2013–2050 were derived from the United Nations (2014) population projections. GDP per capita for 2013–2019 was calculated using forecasts prepared by the International Monetary Fund (2014). 2019 growth rates from this source were applied for years 2020–2050. As for the remaining indicators, a 10-year linear trend of latest data available was used to extend the dataset up to 2050. Average values of the indicators are presented in Fig. 1.

However, even with such a big timeframe, on few occasions a data point for a CEE country had no corresponding value in the dataset for some benchmark countries. In such a case, $\bar{s}_{k}$ from Eq. (1) was capped at 1800 or 2050 (1876 for telephones and 1895 for cars). Meanwhile, the CEE countries dataset, encompassing the years 1920–2012 was not amended to improve coverage, except for creating aggregates for former Czechoslovakia, the USSR and Yugoslavia from data for their successor states.

It should be noted that several countries underwent border changes throughout the timeframe of the study. CEE states were reshaped by the Second World War, chiefly Poland, Romania and the USSR. The data for benchmark countries also reflect post-war adjustments: pre-1918 data for Austria refer to Austro-Hungary (except GDP per capita), pre-1921 data for Ireland cover the whole island rather than the later-independent republic, just as data for the United Kingdom include whole Ireland before 1921 (though health indicators cover only England and Wales before that date). Germany was split into two countries between 1949 and 1990, so an aggregate for both states was used here, except for the number of cars, which refer only to West Germany during those years. Under-reporting for non-white population of United States, Australia, Canada or New Zealand is also present in historical health statistics for these states (Human Mortality Database 2014). None of this issues, however, should have a noticeable impact on the results.

## Results

**Fig. 2:**
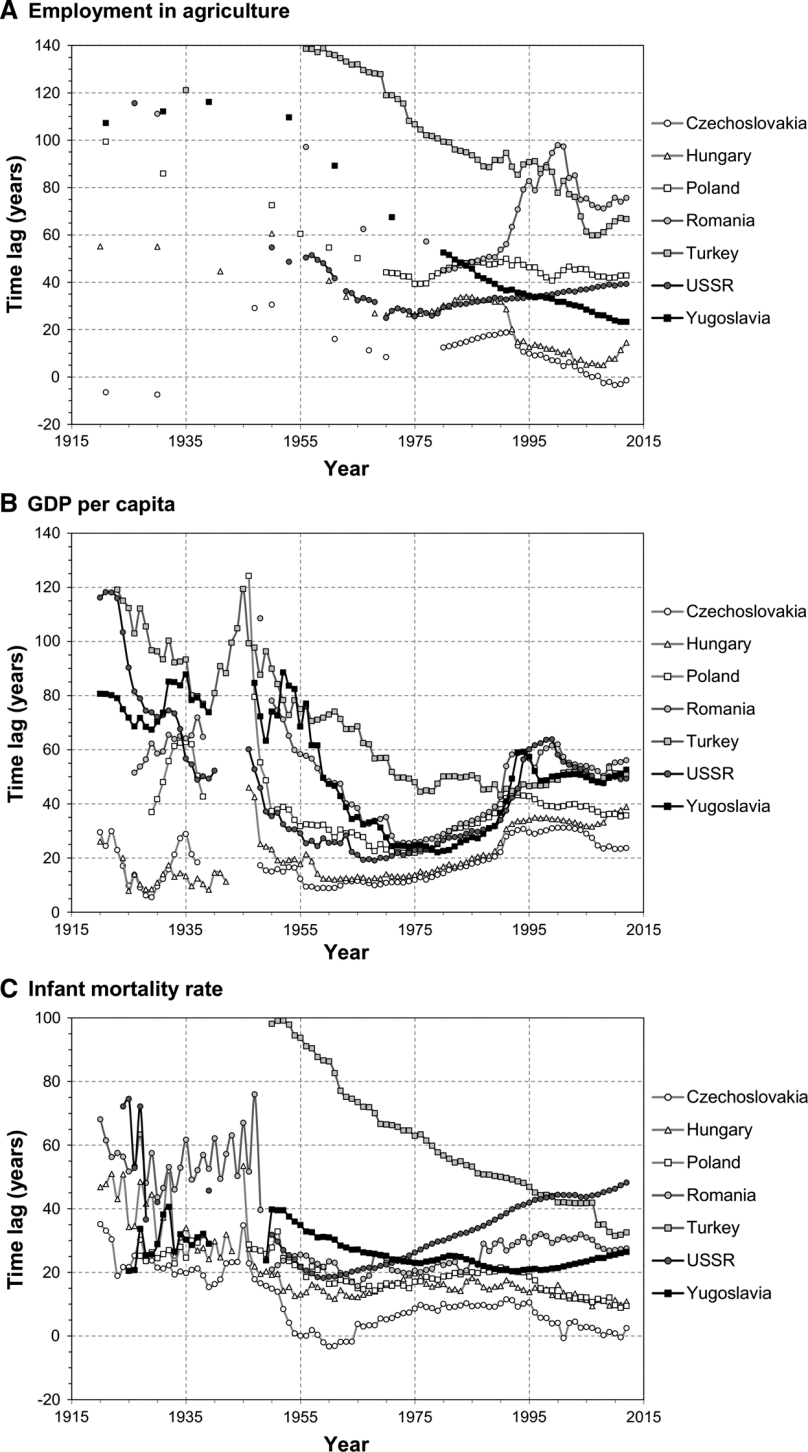

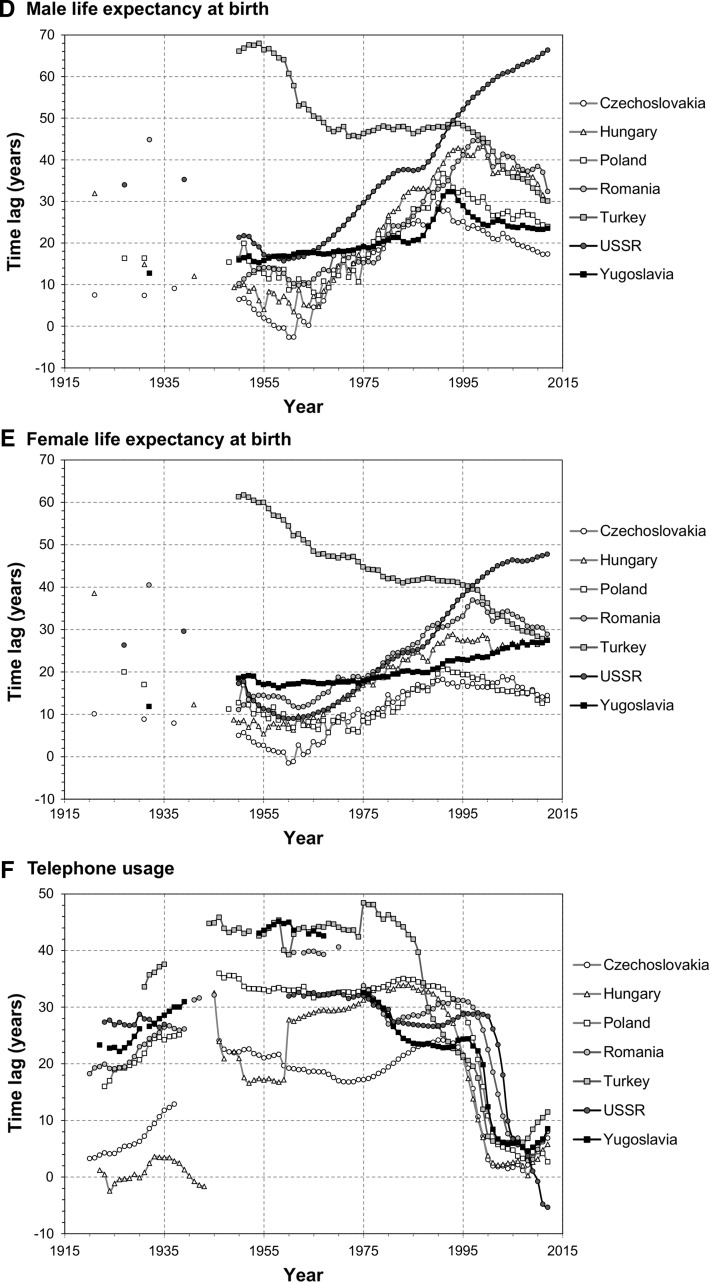

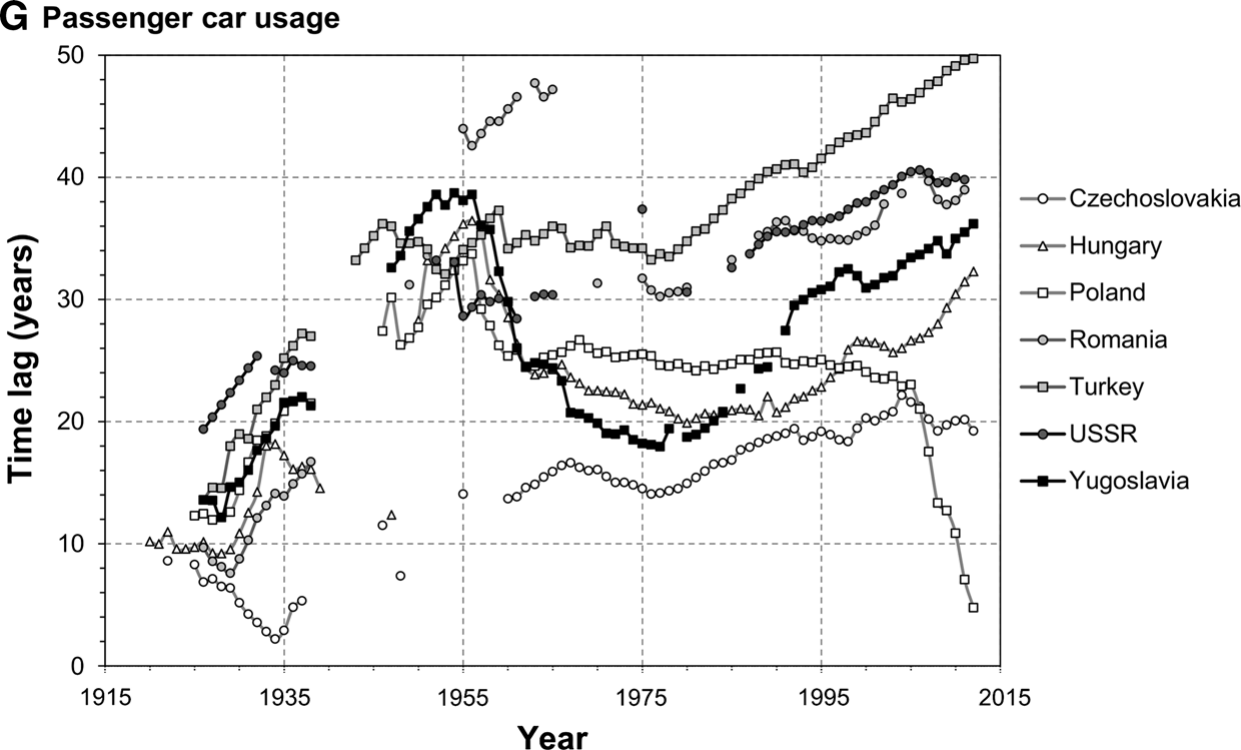
Time lags for selected CEE countries, 1920–2012

The main findings of the analysis are presented in Fig. 2. Only seven of the biggest CEE countries are included in the graphs for the sake of clarity. Results for all countries can be found in the supplementary file.

### Results for Individual Indicators

#### Employment in Agriculture

During the interwar period, around 80 % of the workforce in all bar two CEE countries were employed in the primary sector—a whole century behind the West. Those exceptions were Hungary (50 years) and Czechoslovakia, which actually outperformed the benchmark group. Heavy industrialisation, that took place after the war, resulted in significant progress in a majority of states, especially in Yugoslavia, Bulgaria and Turkey. However, all CEE states except Yugoslavia and Turkey reached their peak in the 1970s (time lag of 10–40 years) and made little progress afterwards. Poland’s time lag of 43 years in 2012 is virtually the same as in 1970. In the former USSR the time lag is steadily increasing since the late 1960s, while Romania also increased its lag since that time. Currently, the Czech Republic and Slovakia are still the only states near the ‘benchmark’ level, while the typical time lag is around 30–50 years; only Albania is still 100 years behind. In general, much convergence with the West could be observed since the 1920s, with an average lag reduced by two-thirds.

#### GDP Per Capita

Progress in this category was much smaller than observed in the previous indicator. In late 1920s, just before the Great Depression, Hungary and Czechoslovakia were only a few years behind the West, while for other states the gap was 40 years and more, up to almost 120 years in the case of Albania. CEE was hit hard by the crisis and even more so by the Second World War. However, despite widespread destruction, the impact of the war was short-lived and the lag in CEE countries reached pre-war levels by the early 1950s. This is partly due to severe drops of GDP per capita in many benchmark states around 1945. Significant convergence was observed later, but it stopped by the early 1970s. More developed states—Czechoslovakia and Hungary recorded their lowest lag in early 1960s and began to slowly diverge from the west. CEE countries mostly recorded a lag of 20–30 years at their peak, though Slovenia (part of Yugoslavia at the time) was only 2 years behind ‘benchmark’ in 1979. Virtually all countries had a lag of around 10 years greater by the time of economic transformation in 1989. That event cost them an additional 10–20 years of development. In 2012, the only countries to have a smaller lag than the minimum during the 1960s–1970s were Albania and Bosnia–Herzegovina. Poland, Estonia and Slovakia are at a similar level compared to 1989, but half of the CEE countries have increased their lag since the transformation by around 20 years. Even Turkey, which did not experience communism, also has a higher lag nowadays than in the 1970s or 1980s. Poland, Hungary and Czechoslovakia were the most developed CEE states in 1920s and made the smallest progress since that time.

#### Infant Mortality Rate

In the 1920s–1930s CEE countries were lagging 20–40 years behind the benchmark states; only Estonia and Latvia were close to Western levels. After the war significant progress was made and by the late 1950s the lag was reduced to 10–20 years, with Czechoslovakia and Baltic states surpassing the benchmark group’s average. However, progress stopped in early 1960s in most states and the lag remained at 15–20 years until the mid-1990s. Only Turkey and Bosnia-Herzegovina recorded steady reduction of lag from the Second World War until today, albeit from a very backward level of more than 90 years in 1950. On the other end of the scale the USSR increased its lag significantly, from 18 years in 1958 to 48 years in 2012. Currently, only Albania lags by more than 30 years, while the Czech Republic and Slovenia surpass the benchmark level by more than 10 years. On the positive side, the whole region recorded smaller lags in 2012 than during the interwar period.

#### Life Expectancy

Before the war most countries lagged by 10–20 years, with negative outliers such as Albania and Romania and positive ones like Latvia and the Czech part of Czechoslovakia. Similarly as infant mortality, the first two decades after World War II were marked by substantial progress. By the late 1950s only Turkey was still lagging by more than 20 years. Bulgaria and Czechoslovakia even exceeded the benchmark level during the 1960s. However, Turkey was the only state to reduce its lag significantly since that period. The gap between communist states and the West widened quickly. By the early 1990s the lag more than tripled, especially in male life expectancy in the USSR, Poland or Romania. The average lag in the region in 1995 was 38 (men) and 27 years (women), in comparison to 11–13 years in the 1960s. Some progress was observed in recent years, mainly in male life expectancy, but the lag is still 30–50 years for males or 20–30 years for females. Especially successor states of the USSR struggle, as their lag increased in the entire Soviet territory from 16 to 66 years (men) and 9 to 48 years (women) between 1960 and 2012. Unlike as in the case of infant mortality, only two states have smaller lags now than before the Second World War (Albania and Turkey).

#### Telephone Usage

The telephone was still a fairly nascent technology in the 1920s; therefore the CEE countries did not lag the benchmark states significantly, mostly below 20 years. But the gap increased in all countries during the interwar period, because CEE states were adopting telephony much slower. Estonia, Latvia and Hungary managed somewhat to keep up with the West, while others were lagging 20 years or more by the late 1930s. A further increase occurred during World War II, due to lack of investment. The time lag remained at the same level in most countries during the subsequent decades. Progress was only recorded in Yugoslavia, Bulgaria and some parts of the USSR. This was changed by the advent of mobile telephony: all CEE states reduced their lags by 20 years in just 5 years. This shift is particularly sharp in the least developed states, like the former Yugoslavia and the USSR. In 2012 the maximum time lag was 11 years (in Albania and Bosnia–Herzegovina), while the regional average is only 3 years. Estonia, Lithuania and Russia are especially keen adopters of mobile phones.

#### Passenger Car Usage

Cars, constructed a decade after the telephone, were a rare item outside North America during the interwar period. As a result, the CEE countries were only 10–20 years behind the benchmark states in the 1920s. However, slow adoption of this technology caused the lag to increase in subsequent years. CEE states have lost most of its vehicle stock during the war and had a lag of 30–40 years in the beginning of the 1950s. Their progress during the next decades differed strongly between them. In Hungary and Yugoslavia, the lag decreased by the late 1970s, but has consistently been rising afterwards. In Turkey and the USSR it changed little until the 1970s, and continues to rise today. In Bulgaria the lag barely changed between 1970 and 2012. In Poland it was even from 1960 until 2005, when it started decreasing rapidly. Also, Lithuania recently experienced a spike in car ownership and has outperformed the benchmark group. This can be attributed to vast imports of used cars from the West after the European Union expanded eastwards in 2004 and, again, in 2007. But the general trend for the region during the entire research period is a continuous increase in time lag, which now stands mostly at 30–40 years.

### General Results

CEE states lag most in development in pure monetary terms, measured by GDP per capita: 50 years on average in 2012, up from 39 years in 1989. The other economic indicator, employment in agriculture, comes second with an average lag of 40 years. Male life expectancy indicates a lag of 34 years, 8 years more than female life expectancy, while progress in reducing infant mortality is much greater (a lag of 15 years on average). While cars are still not that common in CEE states as in the West (29 years behind), telephones already are, since the regional average of time lag in this indicator is only 3 years.

In the light of the above it is obvious that CEE states mainly need to improve their economic performance. If we check what the values were of other indicators in the benchmark countries, when their GDP per capita levels were similar to CEE countries in 2012, the difference is particularly visible. On average, CEE countries have a four times smaller infant mortality rate, a longer life expectancy (men—4 years, women—6 years), almost seven times the number of telephones per 100 persons and nearly double the motorization rate than the benchmark states at a similar GDP per capita level. In other words, all countries, with a few exceptions, are less dependent on agriculture, have healthier populations and use more technology than the West when it was on the same level of economic development. It shows that technology (also medical and agricultural) has become less expensive and widespread, making CEE countries more like their Western counterparts notwithstanding lower incomes. This is in line with findings by Jordá and Sarabia (2014), who found more convergence between the countries in the world in health and education indicators than in income.

Moreover, correlation of lags in income with other indicators is weak. Across the whole sample, the coefficient of determination (R^2^) varies from 0.05 (female life expectancy lag) to 0.43 (employment in agriculture lag). This is significantly less than R^2^ calculated for absolute values of those indicators, which is between 0.25 and 0.69. Correlation between the lags of other indicators is also small, with only two pairs exceeding the R^2^ value of 0.5 (male and female life expectancy lag; employment in agriculture and infant mortality). This contrasts with findings of Comin et al. (2008), which noted a high correlation between income and technology usage lags, as well as between usage of different technologies. The difference can be explained as resulting from the use of a far larger number of countries and a limited historical analysis of that study. Here, weak correlations of lags most likely indicate the importance of policy factors; that the quality of life can be improved without increasing income or that it can stagnate despite a rising GDP per capita.

Over the timespan of the analysis, the CEE states converged little with the West. After a minor reduction of lag during the interwar period, the CEE countries largely closed the development gap between the 1950s and 1970s (Table 2). The war had a small and short-lasting effect on this gap, while the subsequent rapid industrialisation and urbanisation relatively quickly boosted standards of living. However, from around 1970, they are increasingly trailing behind. Falling commodity prices, lack of innovation and the arms race with the West have hampered the Central European economy. The transformation of the 1990s was marked by an extensive restructuring of their economies, which was necessary to balance supply and demand. CEE states have slowly started to converge again in the last decade, powered mainly by the tertiary sector, but their time lag is still mostly larger than the minimum observed about 40 years ago. Unfavourable demographic conditions (shrinking working-age population and significant emigration) and unreformed remnants of the previous economic system put a more rapid convergence with the West at risk.

**Table 2:** Time lags, ca. 1920–2012, average of all indicators

Country	Time lag around the year…										
	1920	1930	1940	1950	1960	1970	1980	1990	2000	2010	2012
Albania							55	61	54	43	43
Bulgaria		46		42	29	20	21	26	30	27	25
Hungary	30	20	18	24	19	19	23	27	23	21	22
Poland		31		33	25	23	27	32	25	20	19
Romania		47		41	37	33	29	37	46	39	38
Turkey				75	69	59	54	49	43	38	38
Former Czechoslovakia	11	7		15	7	12	15	20	15	11	12
Czech Republic								15	11	7	8
Slovakia								23	21	18	19
Former Yugoslavia		40		48	40	30	27	28	28	27	28
Bosnia and Herzegovina									36	34	35
Croatia								20	21	22	22
Macedonia									35	34	36
Serbia with Montenegro								30	37	34	35
Slovenia								14	10	6	5
Former USSR		48	43	32	25	24	29	34	44	41	41
Belarus								30	38	30	30
Estonia							20	27	23	15	13
Latvia							20	25	29	27	27
Lithuania							21	25	28	19	17
Moldova								44	68	54	52
Russia								30	40	32	32
Ukraine								31	46	43	43

As can be seen from the data presented here, adoption of centrally planned economies was unsuccessful in all countries: the time lag at the end of communist rule was mostly the same as near the beginning. Only Turkey, the sole CEE state that did not experience communism, has consistently been reducing its lag, though it used to be the most backward country. Meanwhile, the most developed countries: the Czech Republic and Slovenia are only a few years behind the benchmark group, but they have already been in such a position before the second world war. On the other hand, Bulgaria, Romania or Albania, usually some of the most backward countries, made noticeable progress. This can be partially attributed to the ‘catch-up’ effect, i.e. higher growth rates due to low level of development itself (Barro and Sala-i-Martin 1992), but also to a faster pace of economic and social development in the modern world compared to previous decades and centuries, which could be seen in Fig. 1. Correlations between lags in the interwar or post-war period and modern ones are weak, expect for employment in agriculture and GDP per capita, though the sample size is too small to make more definite conclusions.

### Remarks About the Method

**Fig. 3:**
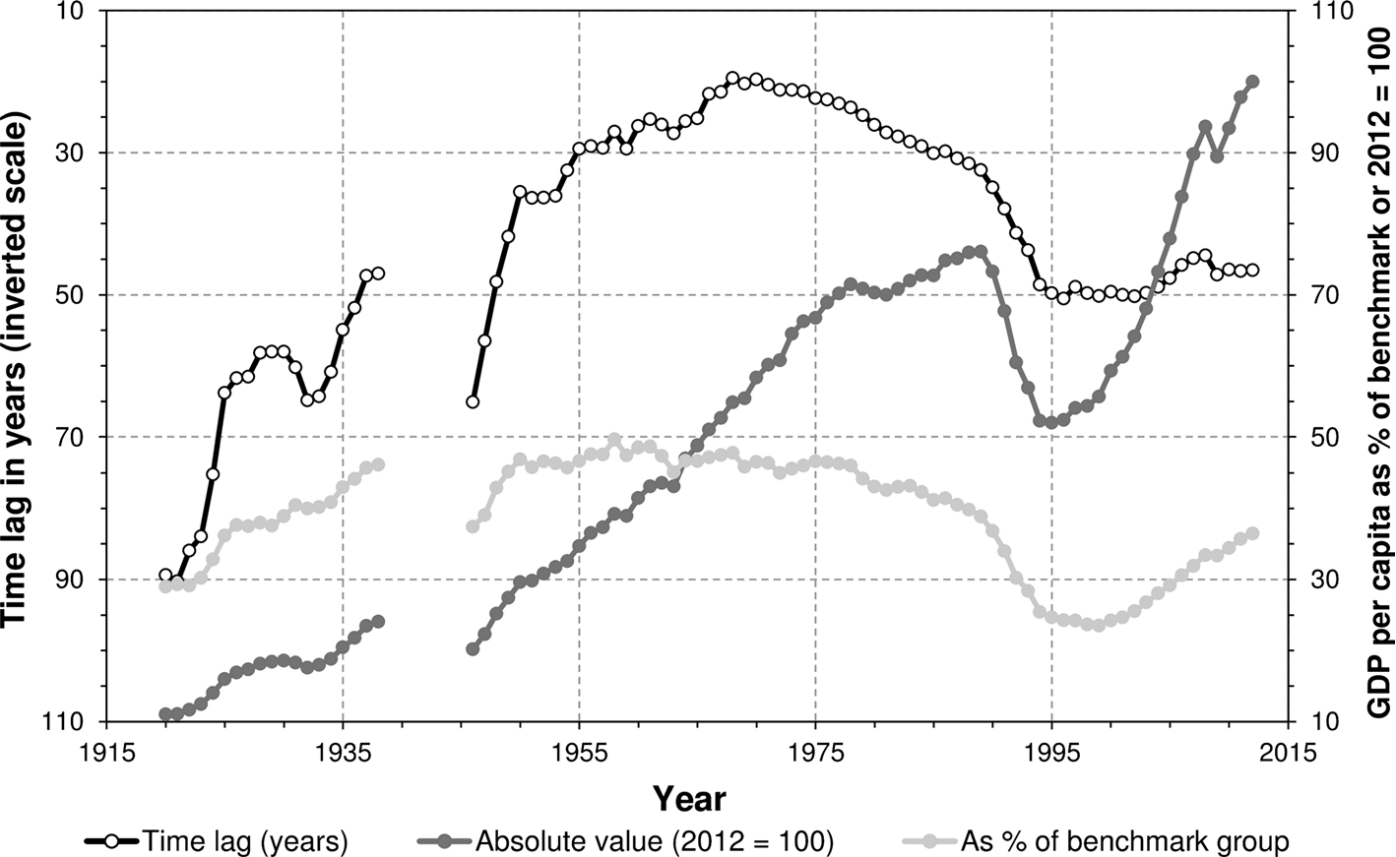
Three measures of GDP per capita for Central and Eastern Europe, 1920–2012

In Fig. 3, three measures of GDP per capita are presented for Central and Eastern Europe as a whole, including a relative indicator mentioned in the introduction. Compared to time lags, relative GDP per capita shows no peak around the year 1970 (when the lag was smallest), while during the early twenty-first century it indicates substantial progress. During 2000–2012 GDP per capita almost doubled, or increased from 24 to 37 % of an arithmetic average of the benchmark group, but the time lag only decreased from 50 to 47 years. The time lags give a different picture of development over time and convergence with richer countries.

The methodology described here could give some useful information about future developments in the CEE states. Although it would not improve the quality of forecasts, it could be helpful to establish realistic development goals based on historical analogies. For example, Hungary will require an average GDP per capita growth rate of 2.4 % per annum for the next 20 years (2012–2032), merely in order to maintain its time lag. During the same period it needs to increase life expectancy by at least 4.2 for males or 3.5 years for females and almost halve its infant mortality rate to keep up with the benchmark states. Such information could be useful for setting achievable and justified goals in all kinds of national or international development strategies. In Poland, a similar document outlines a target of reaching life expectancy of 78 (men) and 84 years (women) by 2030 (Ministry of Administration and Digitization 2013). If those aims were achieved, Poland’s lag in male life expectancy would be reduced to 19.7 years from 23.1 years in 2012 with a slight increase of lag in female life expectancy (13 years instead of 12.6 years in 2012).

The method also has its limitations. It is applicable only to some indicators, which continuously increase or decline with progress. The industrial share in the economy or the energy consumption relative to population no longer do apply in developed countries. Moreover, the method requires large amounts of historical data for countries selected to serve as ‘benchmark’. In this analysis, the dataset stretching back to the year 1800 was at times inadequate to provide enough comparison with least developed states. Occurrence of unique events (especially wars) in the benchmark countries can artificially decrease the lag of the analysed countries.

Additionally, the method does not address one of the primary issues of modern economic research, i.e. inequality in income, health and other aspects of the society. It is caused not only by relative scarcity of relevant data outside some Western states, but also by problems with proper interpretation of the subject. As recently calculated by van Zanden et al. (2014), inequality largely fluctuated over time and the correlation between income and inequality changed from positive in the nineteenth century to negative in modern times. The CEE countries observed lower levels of inequality than the West before, during and after the era of communism. They have hiked only in recent years.

## Conclusions

In this paper, I examined the long-term development of the Central and Eastern European countries by applying the ‘time lag’ method to a set of social and economic indicators. The results show that the countries of Central and Eastern Europe only narrowly converged with a set of 23 highly developed ‘benchmark’ states. GDP per capita is the indicator where this region lags most, 50 years on average in 2012. Employment structure, life expectancy and infant mortality present much smaller lags. Results of individual countries are mixed, but problems with replicating the Western countries’ historical progress in income, life expectancy and motorisation are common. It was also found that GDP per capita lags are weakly correlated with lags in other indicators.

In most CEE countries communist system existed between 1945 and 1989. It aimed to outperform the capitalist West economically and, indeed during the first half of that period they closed some of the development gap with the West. However, during the second half (from late 1960s) they had slid back to the starting point. Even today, they mostly lag more than at the peak, before the fall of the centrally planned economy. The only country that have made consistent progress in reducing its time lag is Turkey; it has never been a communist country, though it also used to be the poorest state in the region. Some ‘catch-up’ effect could be observed, but evidence of this is limited.

The ‘time lag’ method was applied here to compare relative development of countries over time. However, countries could also use this method in order to set a different kind of development goals, i.e. aiming to reduce the time lag rather than reaching an arbitrarily chosen value of an indicator.

### Electronic supplementary material

Below is the link to the electronic supplementary material.

Supplementary material 1 (XLS 284 kb)

## Appendix: Data Sources for Indicators

### GDP Per Capita

Bolt and Van Zanden (2013), Maddison (2010), The Conference Board (2014) and national statistical institutes (NSEs).

### Employment in Agriculture

Eurostat (2014), FAO (2014), League of Nations (1927) and United Nations (1949) yearbooks and subsequent editions, Maddison (2001), Nixon (1938), OECD (2014), Schön and Krantz (2012) and NSEs.

### Infant Mortality Rate, Life Expectancy

Eurostat (2014), League of Nations (1927) and United Nations (1949) yearbooks and subsequent editions, Human Life-Table Database (2014), Human Mortality Database (2014), United Nations (2014), World Bank (2014) and NSEs. Pre-war data of life expectancy for Albania and Romania could be found only as an aggregate for both sexes; it was assumed that the gap in life expectancy between sexes was 4 years, a typical value of the time in the CEE states.

### Telephone Usage

Number of phones was derived from Comin and Hobijn (2009), International Telecommunication Union (2014), Universität Zürich (2013), World Bank (2014) and NSEs; usage was calculated by diving the number of phones by population data extracted from League of Nations (1927) and United Nations (1949) yearbooks and subsequent editions, United Nations (2014), Maddison (2010) and NSEs.

### Passenger Car Usage

Number of cars was derived from Comin and Hobijn (2009), Eurostat (2014), Federal Highway Administration (1995), League of Nations (1927) yearbook and subsequent editions, World Bank (2014) and NSEs; usage was calculated by diving the number of cars by population data from sources mentioned in the previous paragraph. For USA and Japan, which have very narrow definitions of passenger cars, I have summed up several categories of cars in order to come close to the much broader definition used in European countries.

## Footnotes

1 It is assumed that *S* is an uninterrupted annual dataset, which I use here. The mathematical representation shown here was adapted from Comin et al. (2008) and simplified, because their data were not necessarily consecutive observations.

2 Montenegro became independent from Serbia in 2006, but due to data constraints Serbia is analysed here together with Montenegro and the partially-recognized state of Kosovo.

3 For consistency of the analysis, aggregate figures for the former USSR includes its Asian successor states, which are not analysed here separately.

4 Luxembourg is excluded from GDP per capita calculations due to limited availability of data.

5 Particularly useful were the international comparisons sections from Swedish and German statistical yearbooks (Kungliga Statistiska Centralbyrån 1914; Kaiserlichen Statistischen Amt 1904, and subsequent editions).
